# CParty: hierarchically constrained partition function of RNA pseudoknots

**DOI:** 10.1093/bioinformatics/btae748

**Published:** 2024-12-19

**Authors:** Mateo Gray, Luke Trinity, Ulrike Stege, Yann Ponty, Sebastian Will, Hosna Jabbari

**Affiliations:** Department of Biomedical Engineering, University of Alberta, Edmonton, Alberta T6G 1H9, Canada; Department of Computer Science, University of Victoria, Victoria, British Columbia V8P 5C2, Canada; Department of Computer Science, University of Victoria, Victoria, British Columbia V8P 5C2, Canada; Institut Polytechnique de Paris, 91120 Palaiseau, Paris, France; Institut Polytechnique de Paris, 91120 Palaiseau, Paris, France; Department of Biomedical Engineering, University of Alberta, Edmonton, Alberta T6G 1H9, Canada

## Abstract

**Motivation:**

Biologically relevant RNA secondary structures are routinely predicted by efficient dynamic programming algorithms that minimize their free energy. Starting from such algorithms, one can devise partition function algorithms, which enable stochastic perspectives on RNA structure ensembles. As the most prominent example, McCaskill’s partition function algorithm is derived from pseudoknot-free energy minimization. While this algorithm became hugely successful for the analysis of pseudoknot-free RNA structure ensembles, as of yet there exists only one pseudoknotted partition function implementation, which covers only simple pseudoknots and comes with a borderline-prohibitive complexity of $O(n^{5})$ in the RNA length *n*.

**Results:**

Here, we develop a partition function algorithm corresponding to the hierarchical pseudoknot prediction of HFold, which performs exact optimization in a realistic pseudoknot energy model. In consequence, our algorithm CParty carries over HFold’s advantages over classical pseudoknot prediction in characterizing the Boltzmann ensemble at equilibrium. Given an RNA sequence *S and* a pseudoknot-free structure *G*, CParty computes the partition function over all possibly pseudoknotted density-2 structures G∪G′ of *S* that extend the fixed *G* by a disjoint pseudoknot-free structure G′. Thus, CParty follows the common hypothesis of hierarchical pseudoknot formation, where pseudoknots form as tertiary contacts only after a first pseudoknot-free “core” *G* and we call the computed partition function *hierarchically constrained (by G)*. Like HFold, the dynamic programming algorithm CParty is very efficient, achieving the low complexity of the pseudoknot-free algorithm, i.e. cubic time and quadratic space. Finally, by computing pseudoknotted ensemble energies, we unveil kinetics features of a therapeutic target in SARS-CoV-2.

**Availability and implementation:**

CParty is available at https://github.com/HosnaJabbari/CParty.

## 1 Introduction

RNA molecules play a vital role in cellular processes; many possess functional structures (Kozak 2005, Cruz and Westhof 2009, Warf and Berglund 2010, Mortimer *et al.* 2014, Wilson and Lilley 2015). As experimental methods to detect RNA structure are time consuming and costly, computational methods for predicting RNA structure have become indispensable. We focus on the accurate prediction of RNA secondary structure (2D), which in turn sheds light on the 3D structure of the RNA. Various algorithms have been developed to tackle this problem, aiming to predict the most energetically favorable structure based on thermodynamic models and empirical data (Zuker and Stiegler 1981, Rivas and Eddy 1999, Bernhart *et al.* 2008, Reuter and Matthews 2010, Sato *et al.* 2011, Rivas 2020). The best-known, most widely-used thermodynamics-based approaches are the algorithms by Zuker and Stiegler (1981) for predicting RNA secondary structure and due to McCaskill (1990) for computing partition functions.

The Zuker algorithm finds the minimum free energy (MFE) structure among all possible pseudoknot-free structures for the given RNA sequence (Zuker and Stiegler 1981). RNA secondary structure prediction is NP-hard (Akutsu 2000, Lyngsø and Pedersen 2000) and even inapproximable (Sheikh *et al.* 2012) when pseudoknots are allowed. Existing efficient algorithms for exact prediction of pseudoknotted RNA secondary structure handle only restricted classes of structures, trading off run-time and structure complexity (Rivas and Eddy 1999, Reeder and Giegerich 2004, Chen *et al.* 2009b, Jabbari *et al.* 2018).

Offering a stochastic perspective on the entire ensemble of possible pseudoknot-free structures of an RNA, the McCaskill algorithm computes *partition functions*. Algorithmically it has strong parallels to the pseudoknot-free MFE algorithm by Zuker, since both algorithms decompose the same structure space in their dynamic programming scheme. Generally, there is a one-to-one correspondence between the search spaces considered by partition function algorithms, such as McCaskill (1990), and MFE algorithms, provided they are unambiguous and complete (Ponty and Saule 2011). This correspondence also extends to pseudoknotted structure spaces. Consequently, the run-time versus structure complexity tradeoffs that were discussed for pseudoknot MFE algorithms like Chen *et al.* (2009b), Jabbari *et al.* (2018), Rivas and Eddy (1999) are mirrored in (hypothetical) corresponding partition function algorithms. So far, the only pseudoknot partition function algorithm, which realizes this idea, is due to Dirks and Pierce (2003). Their algorithm (D&P) handles the restricted class of simple pseudoknots. While it is implemented in NUPACK, its practical application is limited by the algorithm’s $O(n^{5})$ time and $O(n^{4})$ space complexity.

To address the high time and space complexity of other pseudoknot prediction algorithms, we previously developed HFold (Jabbari *et al.* 2007, 2008). Given an RNA sequence and a pseudoknot-free structure, HFold calculates a potentially pseudoknotted secondary structure with MFE in a full RNA energy model that extends the given structure with, in a specifically defined way, a “compatible” second pseudoknot-free structure. By following this principle, HFold becomes the first MFE algorithm that adheres to the *hierarchical folding hypothesis*. This hypothesis suggests that RNA initially folds into a pseudoknot-free structure, and then additional bases pair to further lower the MFE of the structure, possibly forming pseudoknots (Tinoco and Bustamante 1999). Hierarchical folding has been experimentally observed in the formation of pseudoknotted structures (Cho *et al.* 2009), including frameshift stimulating pseudoknots (Chen *et al.* 2009a).

**Figure 1.**
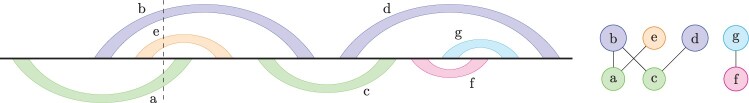
Bands of a bisecondary structure (left) with the corresponding bi-partite crossing graph (right). Note that the connected components of the crossing graph can be understood as groups of bands that directly or transitively cross each other. The structure has density three, since there are positions which are simultaneously covered by three transitively crossing bands. For example, the three bands a, b, and e cover the position indicated by the dashed line. When removing one of the bands a, b, or e, the remaining structure is density-2 (see Section 2). The dynamic programming algorithms CParty and HFold exploit that the crossing bands of density-2 structures are arranged in chains, in the sense that bands a, b, c, d form a 4-chain or f, g form a 2-chain. This allows decomposing density-2 structures by recursively decomposing such chains.

HFold has only cubic time and quadratic space complexity. This means it is as efficient as pseudoknot-free prediction algorithms and, e.g. is faster than CCJ (Chen *et al.* 2009b) or D&P (Dirks and Pierce 2003) by a quadratic factor. HFold takes a pseudoknot-free structure *G* as input, and predicts a pseudoknot-free structure G′ such that G∪G′ has MFE among all *density-2* structures (Jabbari *et al.* 2008) (Fig. 1; Section 2). The class of density-2 structures allows for arbitrary depth and length of nested pseudoknots including H-type pseudoknots and kissing hairpins. This class encompasses structures not handled by CCJ and is more comprehensive than the structure class described by the partition function algorithm by Dirks and Pierce (2003). Since the selection of the non-pseudoknotted partial structure *G* is crucial in hierarchical folding, previous work has identified promising techniques for selecting *G* (Jabbari and Condon 2014, Trinity *et al.* 2023). These techniques involve computing energetically favorable pseudoknot-free structures (Jabbari and Condon 2014) or choosing partial structures compatible with chemical modification data, such as SHAPE reactivity (Trinity *et al.* 2023).

The main objective of this work is to develop and study a partition function counterpart to HFold. To achieve this, we present CParty, a *constrained partition function* (CPF) algorithm that considers possibly pseudoknotted density-2 structures.

The primary challenge in constructing the CParty algorithm is that HFold decomposes density-2 structures in a non-trivially redundant manner. Removing or avoiding such ambiguities is a general and recurring issue in the construction of dynamic programming algorithms to compute RNA partition functions, e.g. McCaskill (1990), Dirks and Pierce (2003). Since partition functions sum over the weights of all considered structures, any ambiguities directly lead to over-“counting”. To address this problem, we have resolved all ambiguities in the decomposition process. This careful preparation step, finally enables deriving the CParty algorithm by a systematic exchange of algebras (e.g. a core idea of ADP (Giegerich 2000)).

Similar to HFold, CParty takes an RNA sequence and an input structure *G*. Then, it calculates the hierarchically CPF over G∪G′, where G′ is pseudoknot-free and $G\cup G^{\prime }$ is density-2. Here, we focus on the algorithm for calculating this hierarchically CPF (i.e. partition function for the ensemble of structures G∪G′, where *G* is fixed) and we leave the base pair probability calculation as a future direction.

To clarify, CParty is designed to compute hierarchically CPFs of RNAs, which ensures very high efficiency. In some applications, this hierarchical approach can be advantageous, as it allows for the integration of prior knowledge. Additionally, its efficiency makes it suitable for iterated use in meta-strategies (cf Jabbari and Condon (2014)).

### 1.1 Contributions

We introduce the novel hierarchical constraint partition function algorithm CParty as a counterpart to HFold. CParty decomposes the density-2 structure class completely and, in contrast to HFold, unambiguously. We implement CParty to perform realistic computations using a full-featured pseudoknot energy model (HotKnots 2.0 (Andronescu *et al.* 2010)), thoroughly scrutinize the implementation, and study its properties. Through empirical time complexity analysis, we demonstrate that CParty outperforms the only other existing pseudoknotted partition function algorithm in NUPACK. Applying our novel tool to the SARS-CoV-2 frameshift element, we compute constrained ensemble energies and unveil a key kinetic transition of its pseudoknot (Kelly *et al.* 2020).

## 2 Materials and methods

### 2.1 RNA secondary structure

An RNA *sequence* of length *n*, known as the *primary structure* of RNA, is represented as a string in ${\left\{A,C,G,U\right\}}^{n}$. Its *secondary structure* is a set of base pairs i.j, where 1≤i<j≤n, and each *base* 1≤i≤n occurs in at most one pair (no triplets). A secondary structure is called *crossing* or *pseudoknotted* if there are at least two base pairs, i.j and i′.j′, that *cross* each other (i.e. i<i′<j<j′ or i′<i<j′<j). Otherwise, it is called *pseudoknot-free* or *non-crossing*. The base pairs i.j and i′.j′ are *nested* if i<i′<j′<j or i′<i<j<j′. Given a secondary structure *G* that pairs *i*, $bp_{G}(i)$ denotes the other end of the base pair of *i* in *G*; similarly, *bp*(*i*) refers to the other end in G∪G′.

#### 2.1.1 Features of pseudoknotted and density-2 structures

Due to space restrictions, we review important features of pseudoknotted structures briefly, and refer to the literature (Dirks and Pierce 2003, Jabbari *et al.* 2008) for full detail; see also Fig. 1. Pseudoknotted structures can be classified by considering specific subsets of base pairs called *bands* (Dirks and Pierce 2003). A band of an RNA structure is a maximal subset of base pairs with the properties that (1) all of its base pairs are pairwisely nested; (2) each base pair of the remaining structure crosses either all or no base pairs of the band; and, moreover, (3) the base pairs of a band cross at least one base pair of the structure.

The literature distinguishes various classes of RNA structures such as simple pseudoknots, kissing hairpins, *k*-knots, and genus *g* that all can be characterized by specific restrictions on the crossing configurations of bands.

In this work, we focus on a specific subclass of *bisecondary* structures (Fontana *et al.* 1993, Haslinger and Stadler 1999, Witwer *et al.* 2004), which can be decomposed into two pseudoknot-free secondary structures. Specifically, we consider the subclass of density-2 structures defined by Jabbari *et al.* (2008) to precisely describe the search space of HFold. Envision the *crossing graph* of a structure that consists of one node for each band and one edge between any pair of crossing bands. In this graph, we can identify connected components of bands, that are in direct or transitive crossing relation to each other. This allows to characterize bisecondary structures graph-theoretically as the structures with bi-partite crossing graphs. In density-*k* structures, the number of bands per connected component that cover a single position is less or equal *k*. For example, in Fig. 1, positions are covered by up to three bands of the connected component {a,b,c,d,e}. Thus, the example shows the bands of a density-3 structure, the density-2 property is violated by bands *a*, *b*, and *e*; e.g. removing band *e* leaves a density-2 structure.

We require additional technical definitions from HFold’s description (Jabbari *et al.* 2008): In density-2 structures, a *region* [i,j] (denoting positions i,i+1,…,j) is *closed*, either if *i* pairs with *j*, or if they are transitively connected due to a chain of crossing bands. In the latter case, *i* and *j* are the left and right ends of a *pseudoloop*, which is closed by base pairs of *i* and *j* as well as the outer base pairs of the other bands in the chain. For example, in Fig. 1, the outermost base pairs of bands *a*, *b*, *c*, and *d* form such a chain and close a pseudoloop. Sec. Supp1. S1 (Supplementary Information) summarizes further definitions.

### 2.2 Energy model

To assess the energy of RNA structures, we distinguish different types of structural elements, called *loops*, i.e. hairpin loops, stacks, bulges, interior loops, or multiloops. Loops are generally defined by their outer and potentially inner closing base pairs (Rastegari and Condon 2007).

Nearest neighbor energy models define the free energy *E*(*G*) of a secondary structure *G* as the sum of the energies of its loops $E(G)=\sum_{L\in G}E^{\text{loop}}(L)$. A prominent example is the Turner 2004 energy model (Turner and Matthews 2010) for pseudoknot-free RNAs, which is used by RNAfold. For pseudoknotted RNAs, Dirks and Pierce (2003) introduced the DP03 energy model, used for pseudoknot prediction in NUPACK; it extends the Turner model by adding penalties for pseudoknots and bands, as well as parameters to score multiloops that “span” a band.

#### 2.2.1 CParty’s energy model and Vienna RNA based implementation

In CParty, we utilize the DP09 energy parameters of HotKnots 2.0, which improve upon the DP03 energy model due to training on known pseudoknotted structures (Andronescu *et al.* 2010). Specific parameters and loop energy functions are provided in Supplementary Table S1. While HFold calculates loop energies based on SimFold (Andronescu *et al.* 2005), CParty uses the Vienna RNA library (Lorenz *et al.* 2011). For this purpose, the energy model parameters were translated to a compatible format, allowing for better interoperability and comparability with the Vienna RNA package. Additionally, our CParty implementation supports hard constraints that restrict the partition functions to structures that leave specified bases unpaired. We note that CParty is limited to the constraints of its energy model and hence, limited to A.U, G.C, and G.U base pairings.

### 2.3 Problem statement: partition functions over density-2 structures

Given an RNA sequence *S*, a pseudoknot-free secondary RNA structure *G*, CParty computes the hierarchically CPF

(1) $$Z_{S}^{G}=\sum_{\underset{\begin{array}{c} G\prime :G\prime \;\text{is}\;\text{secondary}\;\text{structure}\;\text{of}\;S\\ s.t.\;G\cap G\prime =\{\}\;\text{and}\;G\cup G\prime \;\text{is}\;\text{density}-2 \end{array}}{}}\;\text{exp}\;(-E_{S}(G\cup G\prime )/(RT)),$$

where *T* denotes the temperature (e.g. $T=37^{{}^{\circ}}C$) and *R* denotes the universal gas constant ($R\approx 1.987\;{\text{cal}\;K}^{-1}{\;\text{mol}}^{-1}$).

Analogous to the pseudoknot-free partition function developed by McCaskill (1990), this partition function is defined as the sum of *Boltzmann weights* $\text{exp}(-E_{S}(\hat{G})/(RT))$ of RNA structures $\hat{G}$, where *E_S_* computes the RNA energy. Extending this result, our CPF sums over all density-2 structures that are the union of a given (constrained) structure *G* and a secondary structure G′. The energy *E_S_* is evaluated using a pseudoknot energy model (specifically, DP09). Note that this is a true generalization, reducing to the pseudoknot-free partition function of McCaskill when the structure *G* is empty.

Boltzmann weights, B(e):=exp(−e/(RT)), and partition functions have several immediate applications in the description of the potential structures (called *ensemble*) of an RNA at equilibrium. For example, we obtain the *conditional equilibrium probability* of each structure G∪G′: $\mathrm{Pr}(G\cup G\prime |G,S)=B(E(G\cup G\prime ))/Z_{S}^{G}$, and the *ensemble free energy* $E_{S}^{G}=B^{-1}(Z_{S}^{G})=-RT\ln Z_{S}^{G}$ of the constrained ensemble.

### 2.4 The HFold algorithm and its ambiguity

HFold efficiently minimizes the free energy over all density-2 structures G∪G′ that are hierarchically constrained by a given pseudoknot-free structure *G*. Like *G*, G′ must be pseudoknot-free. Energies are defined by a D&P pseudoknot energy model for the given sequence *S*. As a dynamic programming (DP) algorithm, HFold can be fully defined in terms of its recurrences.

HFold computes the total MFE as the entry *W*(1, *n*) of its DP matrix *W*, where *W*(*i*, *j*) denotes the MFE of the subsequence $s_{i}s_{i+1}\ldots s_{j}$. Each *W*(*i*, *j*) is computed using HFold’s *W*-recurrence with the help of additional DP matrices. These matrices store MFEs under specific conditions: e.g. *V*(*i*, *j*) is the MFE over “closed” structures that pair *i* and *j*, WMB(i,j) requires that *i* and *j* are the ends of a *pseudoloop*, and VP(i,j) is the MFE over the loop closed by i.j that spans a band.

Compared to pseudoknot-free prediction algorithms, HFold requires a large number of helper matrices to decompose density-2 structures and optimize correctly in the DP09 model. For instance, it distinguishes pseudoloops with the rightmost band in G′ (WMB′(i,j)), bands in *G* (BE), parts of a multiloop (*WI*), and parts of a multiloop that span a band (WI′).

#### 2.4.1 Ambiguity

**Figure 2.**
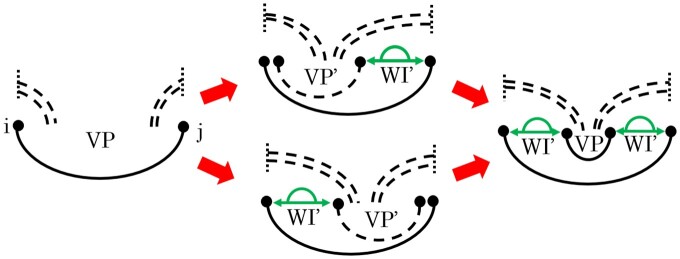
The ambiguity of computing VP(i,j) in HFold. Since i.j is a base pair of a band, it can be crossed by other bands to the left or right (dashed arcs). To handle cases where i.j closes a multiloop that spans the band, HFold utilizes two ambiguous recursion cases (middle) (Jabbari *et al.* 2008) to allow further multiloop branches on the left and/or right of the next base pair of the band. These different cases can converge (right) and produce the same structures in different ways, leading to ambiguity.

Several of HFold’s recurrences are non-trivially ambiguous, preventing a direct translation of the HFold recurrences for CParty. A good example is the decomposition of multiloops spanning a band in the VP recurrence, as illustrated in Fig. 2.

### 2.5 Dataset

For the time and space complexity analysis of CParty we obtained 2808 sequences from the RNASTRAND V2.0 database (Andronescu *et al.* 2008). The smallest sequence has a length of eight nucleotides, while the largest is 1500 nucleotides long. For each sequence, we identified the 20 most stable stem-loop by calculating only hairpin and stacking base pair energies across the whole sequence. These stem-loop structures were then used as constraints.

To assess the impact of constraint variation on CParty, we obtained four sequences of length 968 from the RNASTRAND V2.0 database (Andronescu *et al.* 2008). We generated 24 dinucleotide-shuffled versions for each sequence, resulting in a total of 100 sequences, using the MEME suite (Bailey *et al.* 2015). Using the same method as in the time and space complexity analysis, we generated the 20 most stable stem-loops for each sequence to be used as constraints, as well as the output of RNAfold, for a total of 21 input constraints for each sequence.

## 3 The CParty algorithm

To address the partition function problem corresponding to HFold’s energy minimization problem, we build on HFold’s decomposition of the constrained density-2 structure space. However, the ambiguity in HFold’s decomposition prevents a straightforward rewriting of the energy minimization recurrences into correct partition function recurrences by simply swapping the minimization algebra (min,+) with the “partition function algebra” (+,·). Therefore, as our core contribution, we resolve all these ambiguities by carefully rewriting HFold’s recurrences and introducing new structure classes and recurrences. This enhancement ensures a complete and unambiguous decomposition of the density-2 class of structures.

Here, we discuss the main recurrences of the CParty algorithm and refer readers to the Supplementary Information for a detailed explanation of the remaining recurrences.

### 3.1 General density-2 structures

Corresponding to the *W*(*i*, *j*) recurrence in HFold, $Z_{W}(i,j)$ denotes the partition function over all density-2 structures $R_{i,j}=G_{i,j}\cup G\prime_{i,j}$ for the subsequence $s_{i}\ldots s_{j}$ and input substructure $G_{i,j}$, taken over all choices of $G\prime_{i,j}$.

Call a base *r covered by G*, write isCovered(G,r), iff it is *covered* by some base pair k.ℓ∈G, i.e. k<r<ℓ. Note that $Z_{W}(i,j)$ is defined only for *weakly closed* regions, where no base in the region [i,j] pairs with a base outside of the region. For empty region (*i *>* j*), $Z_{W}(i,j)=1$—accounting for the empty structure with energy 0. Moreover, $Z_{W}(i,j)=0$, if *i* or *j* is covered by *G*

$$Z_{W}(i,j)=\sum \{\begin{array}{c} \left(1)\sum_{\begin{array}{c} \overset{i\leq r<j}{}\\ \bar{\text{is}\;\text{Covered}(G,r)} \end{array}}Z_{W}(i,r-1)\cdot Z_{V}(r,j\right)\\ \left(2)Z_{W}(i,j-1\right)\\ \left(3)\sum_{\begin{array}{c} \overset{i\leq r<j}{}\\ \bar{\text{is}\;\text{Covered}(G,r)} \end{array}}Z_{W}(i,r-1)\cdot Z_{P}(r,j)\cdot B(P_{s}\right) \end{array}$$

**Figure 3.**
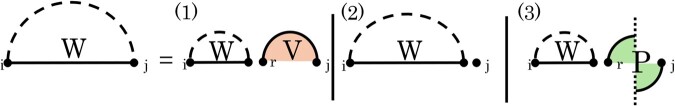
$Z_{W}(i,j)$ recurrence in graphical notation: dashed arcs indicate possible structure, each solid arc represents a base pair. The dotted vertical line indicates an overlapping chain of bands of arbitrary length and that the chain can begin or end via either *G* (above horizontal line) or G′ (below horizontal line). Filled in circles show regions covered by specific structure classes, solid single arc for *Z_V_*, and overlapping chains for *Z_P_*.

Figure 3 illustrates the three cases of *Z_W_*. Case (1) decomposes the structures, where *j* is paired to some *k* in [i,j]; it recurses to $Z_{V}(i,r)$, the partition function over all structures closed by r.j. Case (2) handles structures where *j* is unpaired. Case (3) is analogous to Case (1), but *r* and *j* are left and right ends of a pseudoloop. The case recurses to $Z_{P}(r,j)$ (see below), and penalizes the pseudoknot initiation $(P_{s}).$

### 3.2 Structures closed by a pseudoloop

The partition function over [i,j] where *i* and *j* are ends of a pseudoloop is calculated as $Z_{P}(i,j)$. The decomposition splits of the rightmost band of the pseudoloop with ends *i* and *j*. The band can be in either *G* or G′. We handle the former case in the recurrence of *Z_P_* and the latter in $Z_{PG\prime }$.

**Figure 4.**
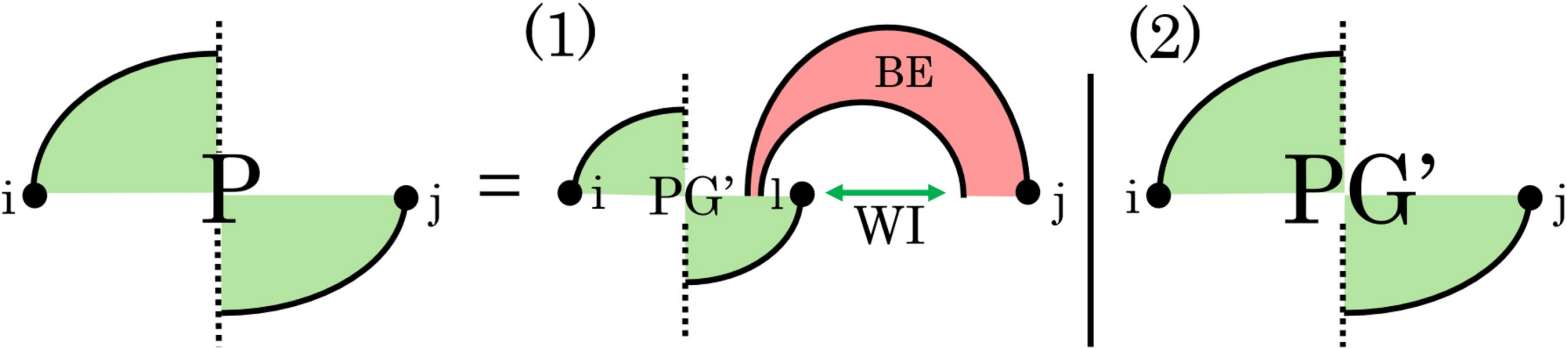
Cases of *Z_P_*. (1) *j* is paired in *G* and there must be some base, *l*, between $bp_{G}(j)$ and *j* that is paired in G′. (2) *j* is not paired in *G*, then move directly to $Z_{PG\prime }$. Filled in circles show regions covered by specific structure classes, non-crossing arcs for $Z_{BE}$, and crossing arcs for *Z_P_* and $Z_{PG\prime }$. Detailed recurrences are provided in the Supplementary Information.

Figure 4 illustrates cases of the *Z_P_* recurrence. The vertical dashed line in the figure symbolizes a series of crossing alternating bands of unspecified length.

We distinguish whether *j* is paired in *G* (Case 1) or in G′ (Case 2). In Case 1, each valid structure must contain a base pair in G′ that crosses $bp_{G}(j)$, where $bp_{G}(j)$ denotes the base pair of *j* in *G*. This forms part of the pseudoloop. We consider all possible choices for the right end of this base pair, denoted as *l*. Each *l* determines unique inner and outer base pairs of the rightmost band (Jabbari *et al.* 2008).

Note that for a given *G*, only one case can be applicable (depending on whether *j* is paired in *G*). To maintain unambiguity, the corresponding sets of structures for different *l* must be disjoint, which is true for density-2 structures. Each single entry of *Z_P_* is computed in linear time, and there is a quadratic number of entries.

### 3.3 Pseudoknotted structures with rightmost band in G′

**Figure 5.**
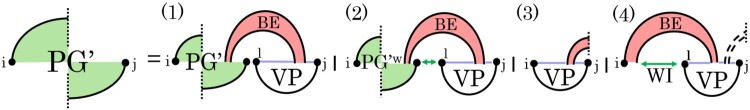
Cases of $Z_{PG\prime }$. (1) handles two rightmost elements of the chain and continues. (2) is similar to (1) except there is a weakly closed region between the bands, this will be handled by $Z_{PG\prime^{w}}$ structure class to preserve the cubic time complexity. For the end cases we have (3) leftmost band of chain in G′; and (4) leftmost band in *G*. Dashed arcs indicate possible structure, each solid arc represents a base pair. Filled in circles show regions covered by specific structure classes, crossing arcs for $Z_{PG\prime^{w}}$. Colored lines correspond with structure classes that may or may not have any substructures: *Z_WI_* showed with double arrows, and light line for $Z_{VP}$. Detailed recurrences are provided in the Supplementary Information.

The partition function of the structures closed by a pseudoloop with ends *i* and *j* and rightmost band in $G^{\prime }$ is calculated in $Z_{PG\prime }$ (Fig. 5).

In case (1), *j* pairs with *l* such that l.j crosses a band of *G*. $Z_{VP}(l,j)$ accounts for the contribution of region closed by l.j, and *Z_BE_* accounts for the contribution of the band in *G*. We then recurse back to $Z_{PG\prime }$ to consider the contribution of the rest of the structure. Case (2) is similar to case (1) with the only difference being the nested substructures allowed between the bands, which is handled by $Z_{PG\prime^{w}}$ in this case. The introduction of $Z_{PG\prime^{w}}(i,j)$ prevents multiple adjacent weakly closed subregions in the pseudoloop.

Cases (3–4) of $Z_{PG^{\prime }}$ are end cases, where only one or two bands, respectively, need to be accounted for. If $i\geq j,\;Z_{PG\prime }=Z_{PG\prime^{w}}=0$.

### 3.4 Structures closed in *G*′, crossing *G*

**Figure 6.**
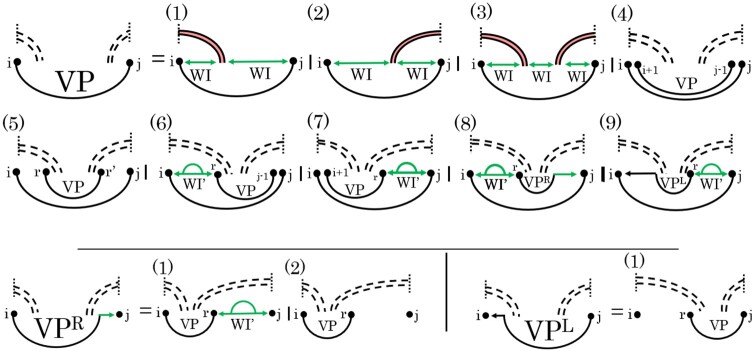
Cases of $VP,\;VP^{R}$, and $VP^{L}$. Top: VP(1−3) either two or three *WI* subregions (green) between *i* and *j*, band regions excluded. (4−5), stacked pair and internal loop, respectively. (6−9), i.j closes a multiloop spanning a band. Bottom-left: $VP^{R}$, i.e. i.bp(i) in G′ crosses base pair in *G*, bp(i)≠j. $VP^{R}$ (1) weakly closed non-empty region [r+1,j], (2) empty region [r+1,j]. Bottom-right: $VP^{L}$, i.e. bp(j).j in $G^{\prime }$ crosses base pair in *G*, bp(j)≠i. $VP^{L}$ (1) empty region [i,r−1]. Dashed arcs indicate possible structure, each solid arc represents a base pair. Colored lines correspond with structure classes: *Z_WI_* showed with double arrows may or may not have any substructure, but for $Z_{WI\prime }$ which also has an arc, there must be some substructure. Detailed recurrences are provided in the Supplementary Information.

$Z_{VP}(i,j)$ is the partition function over all structures $R_{i,j}$ in which i.j∈G′ and crosses a base pair in *G* (Fig. 6). If i≥j, *i* or *j* is paired in *G*, or i.j does not cross any base pair of *G*, then $Z_{VP}(i,j)=0$.

Cases (1–3) of $Z_{VP}(i,j)$ handle nested substructures where there are no other base pairs in [i,j] that cross the same band(s) that i.j crosses. These nested substructures are managed by the WI recurrence (see Supplementary Information). The three cases are disjoint: either *i* is covered in *G* (Case 1), *j* is covered in *G* (Case 2), or both are covered (Case 3). In Case (4), i.j and (i+1).(j−1) form a stacked pair; substructures created by (i+1).(j−1) are handled recursively by $Z_{VP}$. In Case (5), i.j and r.r′ close an internal loop, and we recurse back to $Z_{VP}(r,r\prime )$ for the structures formed by r.r′. Cases (6–9) handle i.j closing a multiloop that spans a band. In these cases, one band of the multiloop crosses the same band in *G* that i.j crosses, and the rest of the multiloop bands and unpaired bases are handled by WI′ recurrences as nested substructures. In Case (6), r.(j−1) crosses the base pair in *G* that i.j crosses, and [i+1,r−1] is a non-empty weakly closed region. In Case (7), (i+1).r crosses the base pair in *G* that i.j crosses, and [r+1,j−1] is a non-empty weakly closed region. In Case (8), [i+1,r−1] is a non-empty weakly closed region, r.bp(r) crosses the base pair in *G* that i.j crosses, and [bp(r)+1,j−1] is weakly closed. We introduce $Z_{VP}^{R}(i,j)$ (see the bottom-left part of Fig. 6), the partition function over all structures such that i.r∈G′ crosses a band in *G*, and r≠j (distinct from Case (6)). Finally, in Case (9), [r+1,j−1] is a non-empty weakly closed region, bp(r).r crosses the base pair in *G* that i.j crosses, and [i+1,bp(r)−1] is empty. We introduce $Z_{VP}^{L}(i,j)$ (see the bottom-right part of Fig. 6), the partition function over all structures such that r.j∈G′ crosses a base pair in *G*, r≠i (distinct from Case (7)), and [i,r−1] is empty.

## 4 Correctness

In the following, we argue that the cases of *Z_W_* fully decompose the density-2 structure class, and are unambiguous. The proof sketch for correctness works by structural induction, showing the correctness of each case.

Theorem 1. *The recurrence of* $Z_{W}(i,j)$*is complete, correct, and unambiguous.*

Recall that $Z_{W}(i,j)$ is the partition function over the set of structures $G_{i,j}\cup G\prime_{i,j}$ for the subsequence $s_{i}\ldots s_{j}$ taken over all choices of $G\prime_{i,j}$ (which is pseudoknot-free, disjoint from $G_{i,j}$, and such that $G_{i,j}\cup G\prime_{i,j}$ is density-2).

By definition of density-2 there are three possible cases, Case (1): *j* pairs with *r*, i≤r<j, such that r.j closes a pseudoknot-free loop, Case (2): *j* is unpaired, or Case (3): *j* is the rightmost end of a chain of crossing base pairs. These cases are disjoint; additionally, if *j* is paired and closes a pseudoknot-free loop, it cannot also be paired in the rightmost band of a pseudoloop. Therefore, the recurrence is unambiguous. Since every density-2 structure falls into one of these three cases, the $Z_{W}(i,j)$ recurrence is complete. Finally, it is correct, since partition functions can be correctly inferred from smaller subproblems (which are correct by induction hypothesis). ■

Similarly, we have constructed each recurrence to be complete and unambiguous by construction. Of particular importance are Cases (6–9) of $Z_{VP}$ that handle a multiloop that spans a band. For a complete decomposition that preserves the $O(n^{3})$ time complexity, $Z_{VP}^{R}$ and $Z_{VP}^{L}$ are introduced asymmetrically such that there is only one possible path to reach each structure. For example, $Z_{VP}$ Case (8) enforces a structure somewhere in the region between *i* and *r*, and moving to $Z_{VP}^{R}$ Case (1) enforces an additional structure in the subregion adjacent to *j*. To compare with $Z_{VP}$ Case (9), similarly we enforce a structure somewhere in the region between *r* and *j*, but moving to $Z_{VP}^{L}$ there is no possible case to introduce an additional structure adjacent to *i*. Thus, we avoid any ambiguity in $Z_{VP}$ decomposition.

## 5 Complexity

Starting with the *Z_W_* recurrence, we observe that its time and space complexity depend on those of *Z_V_* and *Z_P_*. Since *Z_V_* handles pseudoknot-free loops, its time complexity is $O(n^{3})$, and its space complexity is $O(n^{2})$, where *n* is the length of the input sequence.

*Z_P_* deals with pseudoloops. As *Z_P_* matches the WMB recurrence of HFold, and HFold has been proven to have time and space complexities of $O(n^{3})$ and $O(n^{2})$ respectively, the same applies to *Z_P_*. We further empirically verify *Z_P_*’s time and space complexity (see Section 6). Therefore, *Z_W_*’s time and space complexity remain $O(n^{3})$ and $O(n^{2})$, respectively.

Similarly, all other cases remain within the $O(n^{3})$ time and $O(n^{2})$ space complexity. For example, the time complexity for both $Z_{PG\prime }$ and $Z_{PG\prime^{w}}$ is $O(n^{3})$, as both cases involve searching over all values of *l* for a given region [i,j]. The time complexity of $Z_{VP}$ is dominated by the search over the region [i,j] to find the value of *r*, which is also $O(n^{3})$.

## 6 Empirical results

Since CParty solves the conditional partition function for density-2 structures for the first time, it cannot be directly *benchmarked* against existing algorithms. Nevertheless, some comparisons to RNAfold and NUPACK remain meaningful and can provide insights.

### 6.1 CParty and RNAfold compute identical partition functions on non-crossing structures

**Figure 7.**
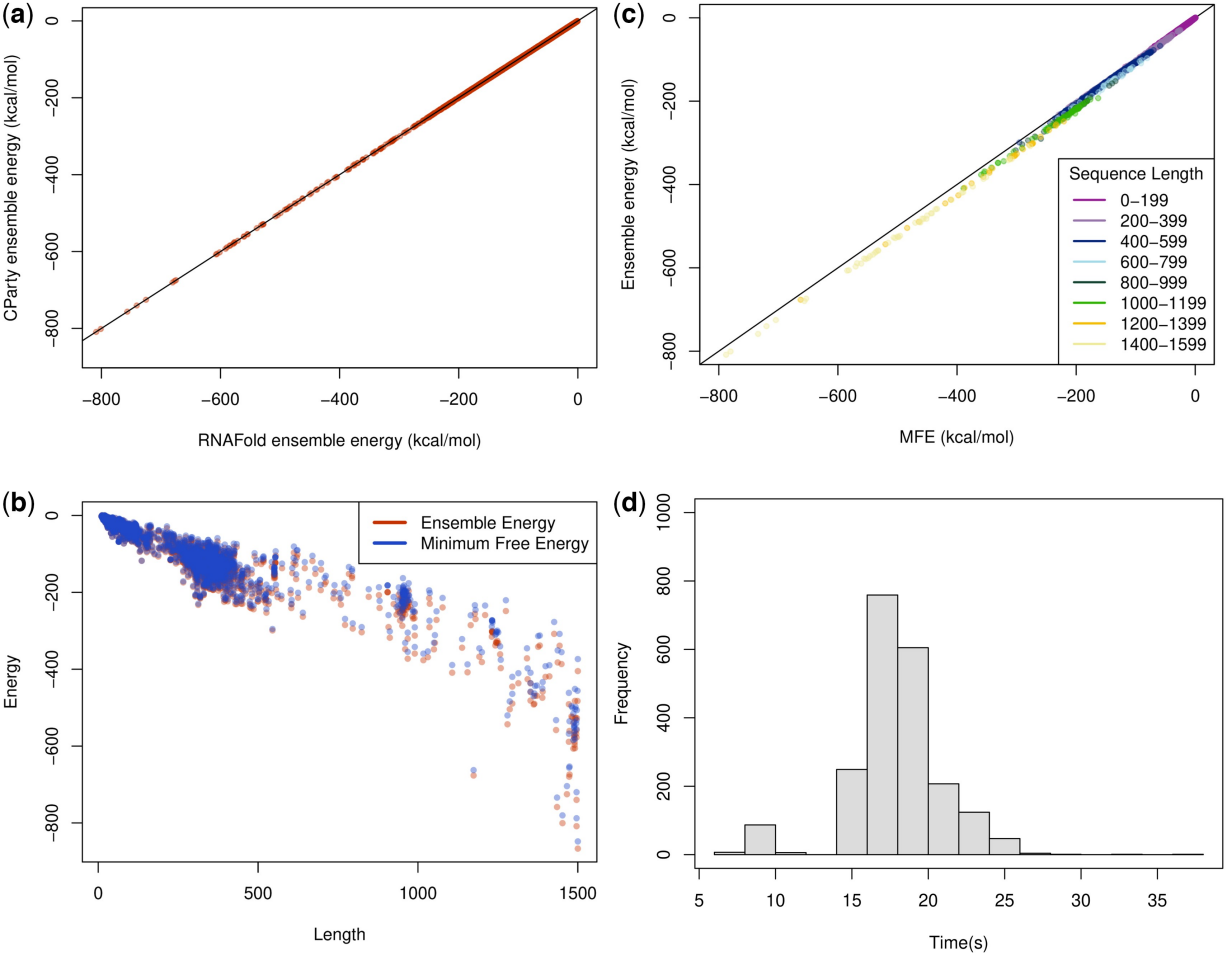
We considered 2733 sequences with length up to 1500 analysed from the RNASTRAND V2.0 database (Andronescu *et al.* 2008). (a) Ensemble free energies without constraints *via*CParty (as the *y*-axis) and RNAfold (as the *x*-axis). Agreement is observed between the two. (b) Ensemble free energies versus minimum free energy of CParty with constraints (pseudoknotted). (c) Each sequence is plotted as its ensemble energy from CParty versus the minimum free energy from HFold. Colors represent the lengths of the sequences. A diagonal line represents a 1 to 1 for ensemble energy to minimum free energy. (d) We plot the results of CParty given a sequence and an input structure. We took four sequences of equal length from the RNASTRAND database (Andronescu *et al.* 2008) and created 24 dinucleotide-shuffled versions for each of them. Each sequence had 21 varying input structures; time was placed in a histogram to show the distribution given different inputs.

Recall that in the special case of an empty input structure *G*, CParty computes a *pseudoknot-free partition function* $Z_{\text{pkfree}}$. As plausibility check, we first compared the ensemble free energy computed by CParty for $Z_{\text{pkfree}}$ to the ensemble free energy for pseudoknot-free structures computed by RNAfold (Lorenz *et al.* 2011). Here, CParty perfectly reproduces the results of RNAfold (Fig. 7a), using Turner 2004 parameters (Mathews *et al.* 2004) without dangle energies.

### 6.2 The MFE variant of CParty resembles HFold

To further validate CParty’s results, we compared CParty-MFE (the MFE variant of CParty) with HFold using 945 density-2 pseudoknotted structures from the RNASTRAND database (Andronescu *et al.* 2008). For each sequence, we extracted a partial structure to use as input. We found that HFold and CParty predicted the same output energy for every sequence and produced identical structures in 913 out of 945 cases. In the remaining cases, the predicted structures differed but resulted in the same energy, representing alternate structures. Both programs were run using the DP09 parameters from HotKnots V2 (Andronescu *et al.* 2010).

These alternate structures are generated due to significant rewrites in both the codebase and the recursions used by CParty. One of the most significant changes is in how CParty traverses the matrix, facilitating the generation of alternative structures. Another major modification is the rewrite of the multiloop recurrence to permit unpaired bases on both sides of a pseudoknot, a feature not allowed in HFold.

### 6.3 CParty does not “invent” pseudoknots in pseudoknot-free RNAs

**Figure 8.**
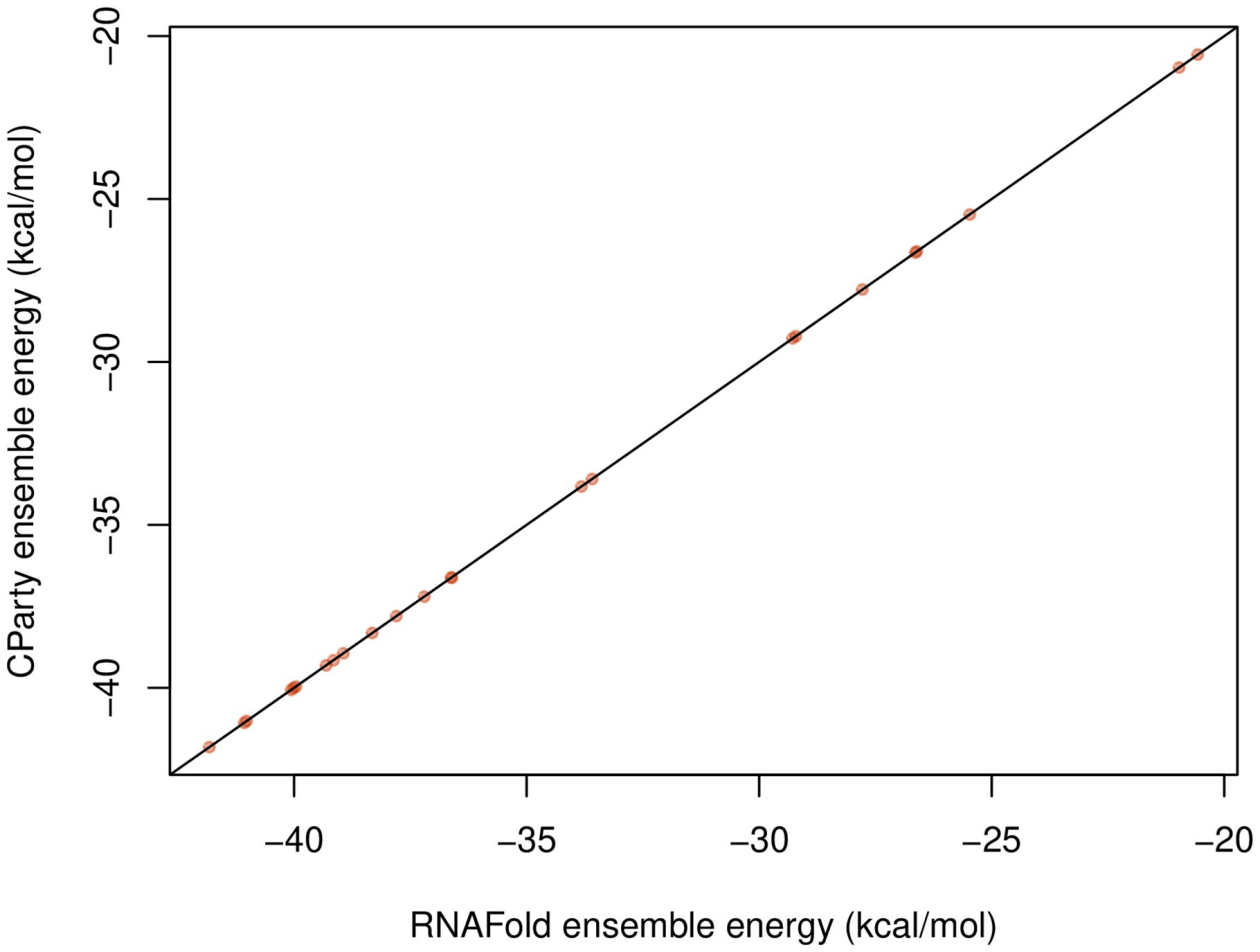
Constrained ensemble free energies by CParty compared to the ones of RNAfold for 24 selected pseudoknot-free tRNAs from RNASTRAND. The virtually identical ensemble energies show that CParty does not predict strong pseudoknotted extensions in its ensemble.

To assess the robustness of CParty against potential mispredictions of pseudoknots, we study the 24 pseudoknot-free tRNA structures from the RNASTRAND database having completely determined sequence and hairpins of at least size 3. For these RNAs, we do not expect energetically strong pseudoknotted extensions of the RNASTRAND reference, which would manifest as differences in the results from CParty and RNAfold. Demonstrating the desirable behavior of CParty, we compare the ensemble energies predicted by CParty and RNAfold, each time constrained by the reference structure, in Fig. 8.

### 6.4 CParty’s empirical time and space outperform NUPACK’s and closely match RNAfold’s

**Figure 9.**
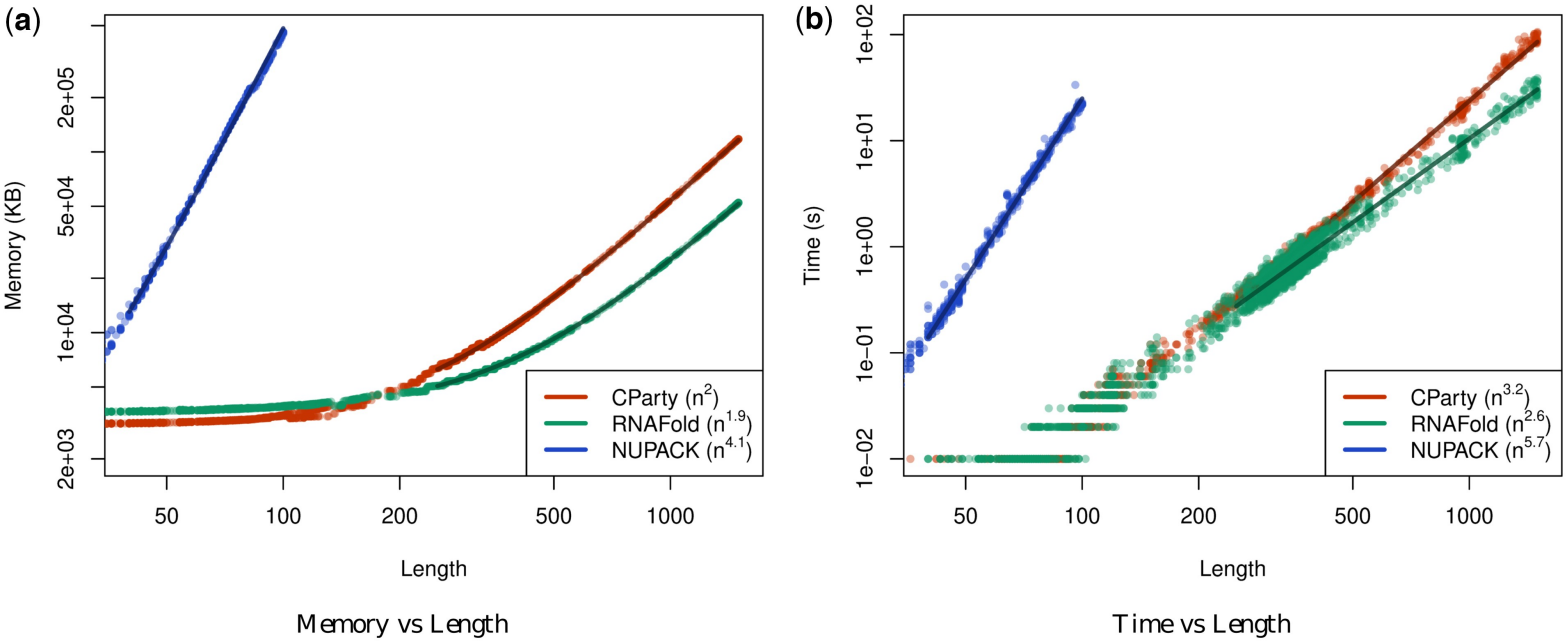
Time and space consumption of CParty versus RNAfold and NUPACK on our dataset, when given an RNA sequence and a pseudoknot-free structure as input. (a) Memory usage (maximum resident set size in KB) versus length (log–log plot) over all benchmark instances. The solid line shows an asymptotic fit $\left(c_{1}+c_{2}n^{x}\right)$ for sequence length *n*, constants *c*_1_, *c*_2_, and exponent *x* for the fit. We ignored all values <250 for CParty and RNAfold and all values <40 for NUPACK. (b) Run-time (s) versus length (log–log plot) over all benchmark instances. For each tool in both plots, we report (in parenthesis) the exponent *x* that we estimated from the benchmark results; it describes the observed complexity as $\Theta (n^{x})$.

We then sought to assess the empirical time and space of computing the CParty partition function, *Z*, against RNAfold and NUPACK. We chose RNAfold as a benchmark for our lower bound and NUPACK as it is the only pseudoknotted partition function calculation algorithm. Since CParty requires an input structure in addition to the RNA sequence, for each sequence we identified the stem-loop structure with the lowest free energy, as detailed in Section 2.5, and used it as input to CParty and RNAfold (NUPACK’s algorithm does not accept a partial structure as input), for a fair run times comparison. All experiments were performed on the Digital Research Alliance of Canada’s Cedar cluster. We measured run-time using user time (see Fig. 9b), and memory using maximum resident set size (see Fig. 9a). The maximum time and memory used by CParty was 103.42 s and 117 212 KB. In comparison, RNAfold had a maximum time of 36.39 s and 52 208 KB. The expected increase in time and space usage when transitioning from pseudoknot-free to pseudoknotted structures in CParty is due to the need for new data structures and additional recurrence relations. As NUPACK requires a large amount of memory, its results were limited to sequences of max length 100. The maximum time and space for NUPACK on this subset of our dataset were 23.05 s and 460 908 KB (see Fig. 9a and b). As seen in Fig. 9, CParty’s time and space complexities closely match those of RNAfold.

### 6.5 Input structure has minimum effect on CParty’s performance

To assess the potential impact of the input structure on the performance of CParty, we calculated the CPF on 100 sequences of length 968 with a total of 2100 various input structures, as detailed in Section 2.5. As shown in Fig. 7d, little variation is observed in memory given different input structures with the 25th and 75th percentiles showing a difference of 10 KB. Figure 7d also provides a median time of 18 s with the 25th and 75th percentiles showing a difference of only 2 s. While variations in CParty’s time and space usage are expected, those were not deemed significant.

### 6.6 Analysis of SARS-CoV-2 frameshift structure

There has been extensive research into predicting structure of the SARS-CoV-2 frameshift sequence, which includes both computational efforts (Schlick *et al.* 2021b, Trinity *et al.* 2023, 2024) and experimental probing experiments (Manfredonia *et al.* 2020, Huston *et al.* 2021, Yang *et al.* 2021, Zhang *et al.* 2021). The frameshift sequence is believed to form a density-2 pseudoknotted structure (Kelly *et al.* 2020, Schlick *et al.* 2021a, Jones and Ferré-D’Amaré 2022).

Employing CParty with different fixed input structures, here we provide a view of suboptimal structures for the SARS-CoV-2 frameshift stimulating structure ensemble. Combining the available SHAPE reactivity probing datasets and various thermodynamic-based algorithms, we previously identified the top-most energetically favorable initial stems for the SARS-CoV-2 77 nucleotide frameshift pseudoknot sequence (Schlick *et al.* 2021b, Trinity *et al.* 2023, 2024). Here, we utilize the top two stems (referred to as initial stems 1 and 2) to explore the structural ensemble for the frameshift sequence. These two stems were identified as pivotal for formation of two of the main structural motifs, referred to as 3_3 and 3_6 (Schlick *et al.* 2021a) (see Fig. 11b).

**Figure 10.**
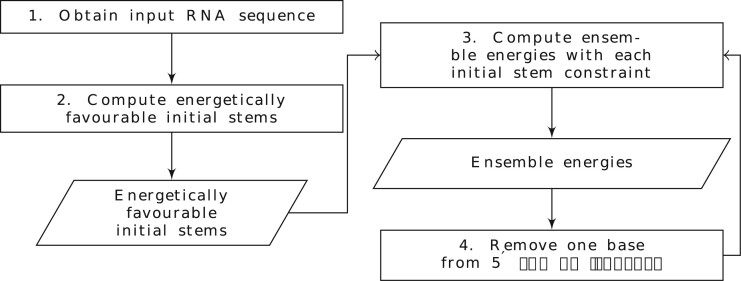
CParty constrained ensemble energy pipeline. Rectangles dictate actions, parallelograms denote outputs.

Following the pipeline of Fig. 10, with each of the two initial stems as constraint, we employ CParty to compute ensemble free energy for sequences of decreasing length (taking seven bases one at a time from the 5′ end), to simulate the effects of the translocating ribosome (Dinman 2012, Atkins *et al.* 2016).

**Figure 11.**
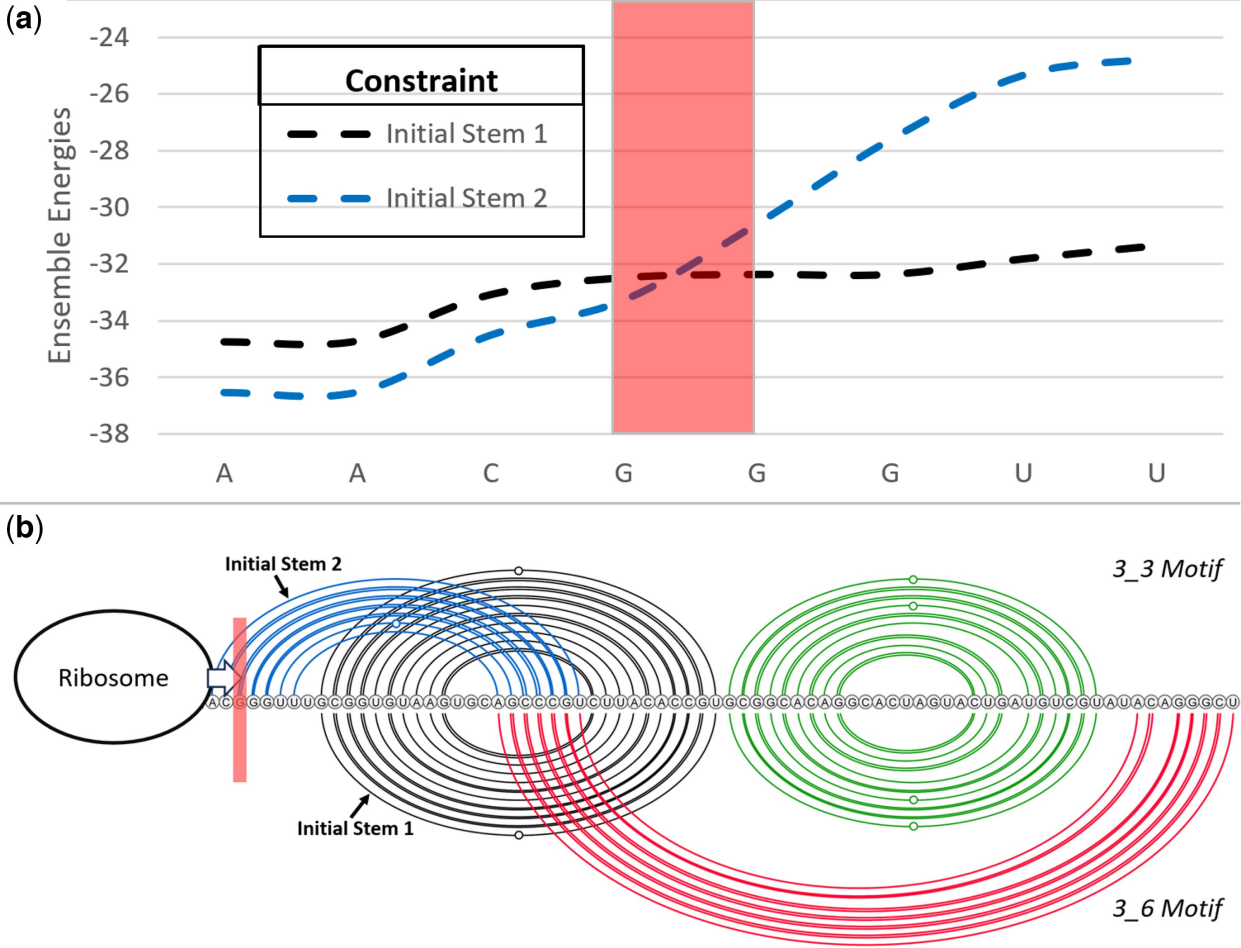
SARS-CoV-2 secondary structure motif transition. (a) Constrained ensemble energies for decreasing SARS-CoV-2 sequence lengths (decreasing from 77 to 70 nt, left to right, sequence labeled on *x*-axis corresponding with 5′ end of RNA strand shown in (b)). (b) Arcs represent base pairs. Initial stem 1 in black (included in both top and bottom pseudoknot motifs), initial stem 2 top 5' end arcs, psuedoknot-free stem top and bottom non-crossing arcs. Top arc diagram: 3_3 motif (Schlick *et al.* 2021a), bottom arc diagram: 3_6 motif (also referred to as the native structure). (a & b): Rectangles highlight the location of a transition from the 3_3 motif to the 3_6 motif. When the ribosome destabilizes the 3_3 motif base pairs (Stem 2) to the left of the rectangle, refolding of the native-type pseudoknot (bottom arcs) is expected.

As seen in Fig. 11, ensemble free energies constrained by initial stems 1 and 2 are close to one another for the 77 length frameshift sequence. However, at the 5th base removal from the 5′ end (see the rectangle in Fig. 11), the ensemble free energy for initial stem 2 increases, suggesting a significant change of structural ensemble at this point. We further investigated this possible structural change using Iterative HFold (Jabbari and Condon 2014); with frameshift sequence and initial stems 1 and 2 as input and decreasing the length by seven bases (one base at a time) from the 5′ end. We noticed that both initial stems 1 and 2 can form the 3_3 structural motif at original sequence length (77). However, at the marked transition form (red rectangle), the 3_3 motif is destabilized while the 3_6 motif maintains its stability. Therefore, the ensemble constrained by initial stem 1 is not affected. This transition observed through both ensemble free energy change as well as structurally supports the hypothesis that destabilization of initial stem 2 *facilitates* subsequent refolding of the native-type pseudoknot (Trinity *et al.* 2024).

## 7 Discussion

In this work, we introduce CParty, a novel biologically motivated algorithm that follows the hierarchical folding hypothesis to efficiently compute the CPF for density-2 RNA secondary structures. CParty takes an RNA sequence and a pseudoknot-free structure *G* as input and computes the CPF over all density-2 structures G∪G′, where G′ is pseudoknot-free and disjoint from *G*.

CParty was developed by addressing the ambiguities in the HFold algorithm (Jabbari *et al.* 2008). While HFold relies on SimFold (Andronescu *et al.* 2005) for pseudoknot-free energy calculations, CParty utilizes the efficient and well-maintained ViennaRNA library (Lorenz *et al.* 2011) and supports various energy models.

CParty handles the class of density-2 structures, which includes a wide range of pseudoknots such as kissing hairpins and interleaved bands of infinite length with arbitrarily nested substructures of the same class. By employing a hierarchical folding approach, CParty achieves a run-time complexity of $O(n^{3})$ and a space complexity of $O(n^{2})$. We evaluated the empirical time and space usage of CParty against RNAfold and NUPACK on a large dataset of RNA sequences of varying lengths, demonstrating CParty’s efficiency in handling large RNA sequences.

Correctly identifying the input structure *G* is an important factor when using our algorithm. As noted in previous studies (Jabbari and Condon 2014, Trinity *et al.* 2023), utilizing the most stable pseudoknot-free stem-loops is effective in identifying both the MFE structure and low-energy suboptimal structures—those energetically close to the MFE structure. Repeatedly sampling the hierarchical distribution with multiple fixed structure choices for *G* can help identify possible folding paths to different secondary structure motifs. Although the input structure influences our algorithm’s run-time and memory usage, we found this impact to be minimal.

In this work, we demonstrated CParty’s application in characterizing structural motifs in the SARS-CoV-2 frameshift element. We believe our algorithm can be similarly used in other structure-function characterizations and aid in the development of novel therapeutics.

Under hierarchical folding assumptions, CParty enables us to calculate the probability of observing a density-2 structure G∪G′ at equilibrium for an RNA *S* as the product of the pseudoknot-free probability of *G* (following McCaskill’s method) and the conditional probability Pr(G∪G′|G,S).

Building on this concept, CParty supports sampling from the corresponding hierarchical structure probability distribution. While we plan to study hierarchical sampling explicitly in future work, it can be achieved through direct stochastic traceback from CParty’s dynamic programming matrices. This process involves a two-step approach: first, sampling pseudoknot-free structures *G* (Ding and Lawrence 2003), and then drawing from the hierarchical distribution constrained by *G*.

By leveraging these capabilities, CParty offers a powerful and efficient method for exploring RNA secondary structures, paving the way for further advancements in RNA research.

## Supplementary Material

btae748_Supplementary_Data

## Data Availability

The data underlying this article are available at https://github.com/HosnaJabbari/CParty.
