# Supplementary material for: CParty: hierarchically constrained partition function of RNA pseudoknots
Source: Bioinformatics. 2024 Dec 19;41(1):btae748. doi: 10.1093/bioinformatics/btae748 (PMC11709253; doi:10.1093/bioinformatics/btae748)
Supplement: btae748_Supplementary_Data [file btae748_supplementary_data.pdf]

1. Supplementary Information

1.1. Definitions

- **RNA molecule**: A sequence of nucleotides, or bases, of length n, of which there are four types: Adenine (A), Guanine (G), Cytosine (C), and Uracil (U).
- **Base pair**: When a RNA folds, bonds form between the bases of the molecule, where each base may pair with at most one other base.
- **RNA structure $\mathbf{R}$**: The set of base pairs $i.j$, $1 \leq i < j \leq n$ such that no index occurs in more than one base pair, and each base pair is one of the canonical base pairs: $\{A-U, C-G, G-U\}$.
- $\mathbf{bp_R(i)}$: We let $bp_R(i)$ denote the index of the base that is paired with base i in R, if any.
- **cross**: if $i.j$, $i'.j'$, and $i < i' < j < j'$, we say that the pair $i.j$ crosses the pair $i'.j'$ (and $i'.j'$ crosses $i.j$)
- **Pseudoknotted base pair**: We say that i.j is a pseudoknotted base pair if for some other base pair i'.j' in R, i.j crosses i'.j'.
- **Pseudoknot-free structure**: If there are no pseudoknotted base pairs in the given structure, it is called a pseudoknot-free secondary structure.
- **Cover**: Let G be a pseudoknot-free structure. Base pair $i.j$ *covers* base k if $i < k < j$ and there is no other base pair $i'.j'$ where $i < i' < k < j' < j$.
- **is_Covered(G,k)**: true iff some base pair of G covers k.
- **Region** $[i, j]$: Sequence of indices between i and j inclusive.
- **Disjoint region**: two regions $[i, j]$ and $[i', j']$ are disjoint if no index is in both regions, i.e $j < i'$ or $j' < i$
- **Weakly closed region**: A region is weakly closed if no base connects a base in the region to a base outside the region.
- **Closed region**: A weakly closed region with at least two bases,$[i, j]$, is closed, if it cannot be partitioned into two smaller weakly closed regions. Note that if $[i, j]$ is closed, then both i and j must be paired, although not necessarily with each other (Rastegari and Condon, 2007).
- **Pseudoknotted closed region**: a closed region $[i, j]$ of a structure R such that $i.bp_R(i)$ and $bp_R(j).j$ are pseudoknotted base pairs.
- **directly banded in**: For a pseudoknotted base pair $i.j$, we say $i.j$ is *directly banded in* $i'.j'$, denoted $i.j \preceq i'.j'$, if $i' \leq i < j \leq j'$ and $[i'+1, i-1]$ and $[j+1, j'-1]$ are weakly closed regions
- **Band**: Consider a maximal chain of $\preceq$. The minimum (maximum) base pair in the maximal chain is the band's inner (outer) closing pair. If $i.j$ is the outer and $i'.j'$ the inner closing pair of a band, then $[i, i']$ and $[j', j]$ are the band's regions
- **Pseudoloop**: Let $[i, j]$ be a pseudoknotted closed region. Then the unpaired bases and base pairs associated with $[i, j]$, together with the closing base pairs of the band associated with $[i, j]$, are members of a *pseudoloop*. The base pairs $i.bp_R(i)$ and $bp_R(j).j$ are the closing base pairs of the pseudoloop.
- **Bi-secondary structure**: A structure R that can be formed by the union of two disjoint pseudoknot-free secondary structures (Witwer et al., 2004).
- **Density**: We define density as follows: Let L be a pseudoloop and $i.bp_R(i)$ and $bp_R(j).j$ be the closing base pairs of L. Let #B(L,k) be the number of bands associated with L that cross k. Then the density of L is the max #B(L,k) for all k in region $[i, j]$. The density of a structure, R, is the maximum density of L over all pseudoloops L of R. We say R is a density-2 structure if the density of R is at most 2.

We provide Fig. 1 and Fig. 2 for density-2 structure class intuition.

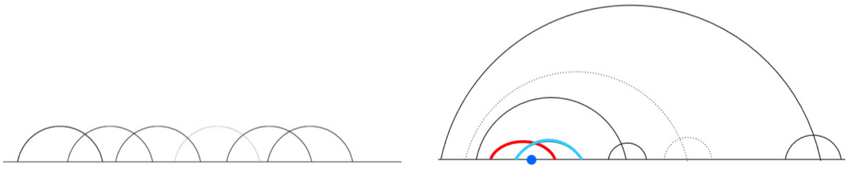

Fig. 1: Arc diagram representation of two density-2 structures, each structure contains an arbitrary number and depth of bands (Jabbari et al., 2008). Blue dot covered by red and light blue bands indicates how association with closed region maintains the density-2 property. Figure modified from (Jabbari et al., 2008).

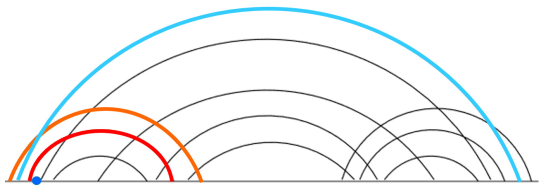

Fig. 2: Example of a *bi-secondary structure* that is not a density-2 structure (Jabbari et al., 2008). Blue dot covered by red, orange, and light blue bands indicate density-2 property not satisfied. Figure modified from (Jabbari et al., 2008).

Table 1. ENERGY PARAMETERS. All parameters were derived at 37 degrees celsius and 1 M salt (NaCl) concentration or extrapolated from experimental values cf. (Jabbari et al., 2008; Andronescu et al., 2010; Ren et al., 2005).

Name	*Description*	*Value (kcal/mol)*
P_s	Exterior pseudoloop initiation penalty	-1.38
P_{sm}	Penalty for introducing pseudoknot inside a multiloop	10.07
P_{sp}	Penalty for introduce pseudoknot inside a pseudoloop	15.00
P_b	Band penalty	2.46
P_{up}	Penalty for unpaired base in a pseudoloop	0.06
P_{ps}	Penalty for closed subregion inside a pseudoloop	0.96
$e_H(i,j)$	Energy of a hairpin loop closed by $i.j$	
$e_S(i,i+1,j-1,j)$	Energy of a stacked pair closed by $i.j$	
$e_{stP}(i,i+1,j-1,j)$	Energy of a stacked pair that spans a band	$0.89 \times e_S(i,j)$
$e_{int}(i,r,r',j)$	Energy of a pseudoknot-free internal loop	
$e_{intP}(i,r,r',j)$	Energy of an internal loop that spans a band	$0.74 \times e_{int}(i,d,e,j)$
a	Multiloop initiation penalty	3.39
b	Multiloop base pair penalty	0.03
c	Penalty for unpaired base in a multiloop	0.02
a'	Penalty for introducing a multiloop that spans a band	3.41
b'	Base pair penalty for a multiloop that spans a band	0.56
c'	Penalty for unpaired base in a multiloop that spans a band	0.12

1.2. Pseudoknotted density-2 structures

Consider the class of secondary structures $G_{ij} \cup G'_{ij}$ that contain pseudoknotted base pairs and cannot be partitioned into two independent substructures for two regions $[i,r]$ and $[r+1,j]$, for some r. Here, we define $Z_P(i,j)$, the partition function over $[i,j]$ when $[i,j]$ is a pseudoknotted closed region, containing a chain of two or more successively overlapping bands that must alternate between $G_{i,j}$ and $G'_{i,j}$, possibly with nested substructures interspersed throughout.

Note that the rightmost band of the pseudoloop may be in G or G'. In order to calculate the energies of substructures in such a structure in our recurrences, we use additional terms including the following: Z_{BE}, Z_{VP}, and Z_{WI}. Roughly, these account for energies of bands spanned by base pairs of $G_{i,j}$, regions enclosed by pseudoknotted base pairs of $G'_{i,j}$ (excluding part of those regions that are within a band of $G_{i,j}$), and nested, weakly closed regions, respectively.

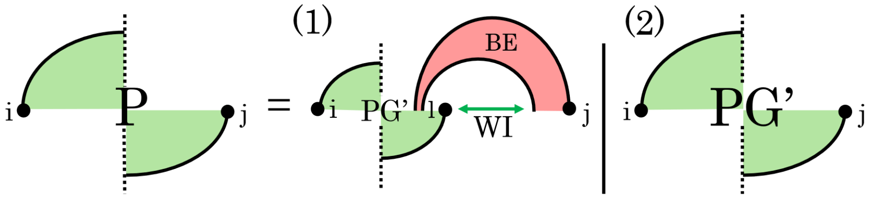

Fig. 3: Cases of Z_P. (1) j is paired in G and there must be some base, l, between $bp_G(j)$ and j that is paired in G'. (2) j is not paired in G, then move directly to $Z_{PG'}$. Filled in circles show regions covered by specific structure classes, red for Z_{BE}, and green for Z_P and $Z_{PG'}$. Detailed recurrences are provided in the appendix.

For $Z_P(i,j)$ base case, if $i \geq j$, then $Z_P(i,j) = 0$, since the substructure is empty, and thus cannot be pseudoknotted. Otherwise, there are two cases (cf. Fig. 3), Case (1): j is paired in G, or Case (2): j is not paired in G. If j is paired in G, then in the MFE structure, some base l with $bp_G(j) < l < j$ must be paired in G', causing $bp_G(j).j$ to be pseudoknotted. We consider all possible choices of l. Once l is fixed, the inner base pair of the band whose outer base pair is $bp_G(j).j$ is also determined (e.g., $b_{(i,l)}$ or $b'_{(i,l)}$ cf. (Jabbari et al., 2008)). The $B(P_b)$ and Z_{BE} terms in Case (1) account for band energy, a Z_{WI} term accounts for the energy of a weakly closed region that is nested in the band, and the remaining energy is represented by the $Z_{PG'}$ term. If j forms a pseudoloop with i and is not paired in G, we move to $Z_{PG'}$ Case (2), partition function over pseudoknotted density-2 structures with rightmost band in G'. Here we note these two cases are disjoint and Z_P is unambiguous, with j either paired in G or G'. Conditions for terms in equation summation are shown in blue.

$$Z_P(i,j) = \sum \begin{cases} (1) \sum_{\substack{bp_G(j)<l<j \\ bp_G(l)=0}} Z_{BE}(bp_G(j), bp_G(B'_{(i,l)})) \cdot Z_{PG'}(i,l) \cdot Z_{WI}(l+1, bp_G(b'_{(i,l)})-1) \cdot B(P_b) \\ (2) \quad Z_{PG'}(i,j) \end{cases} \tag{ii}$$

1.3. Pseudoknotted structures with rightmost band in G'

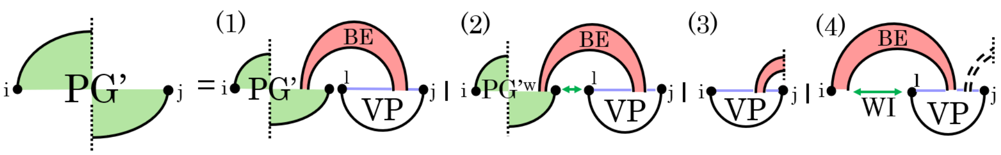

Fig. 4: Cases of $Z_{PG'}$. (1) handles two rightmost elements of the chain and continues. (2) is similar to (1) except there is a weakly closed region between the bands, this will be handled by $Z_{PG'^w}$ structure class to preserve the cubic time complexity. For the end cases we have (3) leftmost band of chain in G'; and (4) leftmost band in G. Dashed arcs indicate possible structure, each solid arc represents a base pair. Filled in circles show regions covered by specific structure classes, green for $Z_{PG'^w}$. Colored lines correspond with structure classes that may or may not have any substructures: Z_{WI} in green, and purple for Z_{VP}. Detailed recurrences are provided in the appendix.

When i and j form a pseudoloop where the rightmost band of the pseudoloop is not in G, i.e., $bp_G(j) = 0$, it must be part of the G' structure. $Z_{PG'}$ handles energy of all structures where the rightmost band is not in G, but is part of the structure G'. Therefore, Cases $(1-2)$ of $Z_{PG'}$ (cf. Fig. 4) are used to account for the energy of the region spanned by the rightmost two bands using $B(2P_b)$, Z_{BE} and $Z_{VP}(l,j)$; and recursively calling either $Z_{PG'}$, or to $Z_{PG'^w}$ if there is a weakly closed region between the bands. To avoid allowing multiple adjacent weakly closed subregions in the pseudoloop, we must introduce $Z_{PG'^w}(i,j)$, the partition function over all pseudoknotted density-2 structures between i and j where the rightmost band of the pseudoloop is part of the structure of G' and the structure is weakly closed. For band border details see (Jabbari et al., 2008). $Z_{PG'}$ Cases $(3-4)$ are end cases, where only one or two bands, respectively, need to be accounted for so no recursive call is made. For the base case when $i \geq j$, $Z_{PG'} = Z_{PG'^w} = 0$.

=

$$Z_{PG'}(i,j) = \sum \begin{cases} (1) \text{ if } bp_G(j) = 0 \\ \quad \sum_{\substack{i<l<b_{(i,l)} \\ isCovered(G_{i,j},l)}} Z_{BE}(bp_G(B_{(i,l)}), bp_G(B'_{(i,l)})) \cdot Z_{PG'}(i,l-1) \cdot Z_{VP}(l,j) \cdot B(2P_b) \\ (2) \text{ if } bp_G(j) = 0, \text{ and } bp_G(l-1) < (l-1) \\ \quad \sum_{\substack{i<l<b_{(i,l)} \\ isCovered(G_{i,j},l)}} Z_{BE}(bp_G(B_{(i,l)}), bp_G(B'_{(i,l)})) \cdot Z_{PG'^w}(i,l-1) \cdot Z_{VP}(l,j) \cdot B(2P_b) \\ (3) \ Z_{VP}(i,j) \cdot B(P_b) \\ (4) \text{ if } bp_G(j) = 0, \text{ and } bp_G(i) > 0 \\ \quad \sum_{i<l<bp_G(i)} Z_{BE}(b_{(i,l)}, b'_{(i,l)}) \cdot Z_{WI}(b'_{(i,l)}+1, l-1) \cdot Z_{VP}(l,j) \cdot B(2P_b) \end{cases} \tag{iii}$$

$$Z_{PG'^w}(i,j) = \sum_{\substack{i<l<j \\ cover(l)=cover(j)}} Z_{PG'}(i,l) \cdot Z_{WI}(l+1,j) \tag{iv}$$

1.4. Structures closed in G', crossing G

$Z_{VP}(i,j)$ is the partition function over all structures $R_{i,j}$ in which $i.j \in G'$ and crosses a base pair in G. The energy of $R_{i,j}$ is the energy of all loops within $R_{i,j}$ that are not inside a band whose base pairs are in G and which crosses $i.j$. If $i \geq j$, i or j is paired in G', or $i.j$ does not cross any base pair of G, then $Z_{VP}(i,j) = 0$, otherwise $Z_{VP}(i,j)$ is computed as follows.

$Z_{VP}(i,j)$ Cases $(1-3)$ handle structures where there are no other base pairs in $R_{i,j}$ that cross the band(s) $i.j$ crosses. These cases are unambiguous: either i is covered, j is covered, or both. In Case (4), $(i+1).(j-1)$ forms a stacked pair (with its energy computed through e_{stP}). In Case (5), $i.j$ and $r.r'$ close an internal loop (with its energy computed through e_{intP}). In Case (6), $r.(j-1)$ crosses a base pair in G and $[i+1, r-1]$ is a weakly closed non-empty region (multiloop spanning band initiation, base pair, and unpaired base penalties, a', b', and c', respectively). In Case (7), $(i+1).r$ crosses a base pair in G and $[r+1, j-1]$ is a weakly closed non-empty region. In Case (8), $[i+1, r-1]$ is a weakly closed non-empty region, $r.bp(r)$ crosses a base pair in G, and $[bp(r)+1, j-1]$ is either empty or non-empty and weakly closed. Therefore, we introduce Z_{VP^R} (cf. Fig. 5), the partition function over all structures such that $i.bp(i) \in G'$ crosses base pair in G, and $bp(i) \neq j$ (distinct from Case (6)). Finally, in Case (9), $[r+1, j-1]$ is a weakly closed non-empty region, $r.bp(r)$ crosses a base pair in G, and $[i+1, bp(r)-1]$ is empty. Therefore, we introduce Z_{VP^L}, the partition function over all structures such that $bp(j).j \in G'$ crosses base pair in G, $bp(j) \neq i$ (distinct from Case (7)), and $[i+1, bp(j)-1]$ is empty.

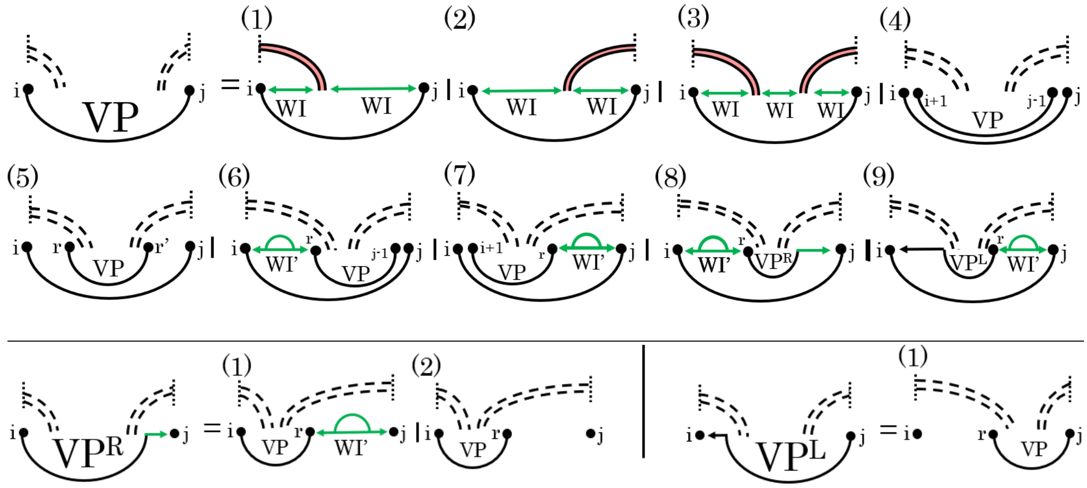

Fig. 5: Cases of VP, VP^R, and VP^L. Top: VP $(1-3)$ either two or three WI subregions (green) between i and j, band regions excluded. $(4-5)$, stacked pair and internal loop, respectively. $(6-9)$, $i.j$ closes a multiloop spanning a band. Bottom-left: VP^R, i.e., $i.bp(i)$ in G' crosses base pair in G, $bp(i) \neq j$. VP^R (1) weakly closed non-empty region $[r+1, j]$, (2) empty region $[r+1, j]$. Bottom-right: VP^L, i.e., $bp(j).j$ in G' crosses base pair in G, $bp(j) \neq i$. VP^L (1) empty region $[i, r-1]$. Dashed arcs indicate possible structure, each solid arc represents a base pair. Colored lines correspond with structure classes: Z_{WI} in green may or may not have any substructure, but for $Z_{WI'}$ which also has a green arc, there must be some substructure. Detailed recurrences are provided in the appendix.

$$Z_{VP}(i,j) = \sum \begin{cases} (1) \text{ if } cover(i) \neq cover(j) \\ \quad Z_{WI}(i+1, B'_{(i,j)}-1) \cdot Z_{WI}(B_{(i,j)}+1, j-1) \\ (2) \text{ if } cover(i) \neq cover(j) \\ \quad Z_{WI}(i+1, b_{(i,j)}-1) \cdot Z_{WI}(b'_{(i,j)}+1, j-1) \\ (3) \text{ if } cover(i) \neq cover(j) \\ \quad Z_{WI}(i+1, (B'_{(i,j)}-1)) \cdot Z_{WI}(B_{(i,j)}+1, b_{(i,j)}-1) \cdot Z_{WI}(b'_{(i,j)}+1, j-1) \\ (4) \text{ if } (bp_G(i+1)=0, \text{ and } bp_G(j-1)=0) \\ \quad B(e_{stP}(i, i+1, j-1, j)) \cdot Z_{VP}(i+1, j-1) \\ (5) \text{ if } cover(G,i) = cover(G,r) \text{ and } cover(G,j) = cover(G,r') \\ \quad \sum\limits_{\substack{i<r<\min(B'_{(i,j)}, b_{(i,j)}) \\ \max(b'_{(i,j)}, B_{(i,j)})<r'<j}} B(e_{intP}(i,r,r',j)) \cdot Z_{VP}(r,r') \\ (6) \sum\limits_{\substack{i<r<\min(B'_{(i,j)}, b_{(i,j)}) \\ bp_G(r)=0}} Z_{WI'}(i+1, r-1) \cdot Z_{VP}(r, j-1) \cdot B(a'+2b') \\ (7) \sum\limits_{\substack{max(B_{(i,j)}, b'_{(i,j)})<r<j \\ bp_G(r)=0}} Z_{VP}(i+1, r) \cdot Z_{WI'}(r+1, j-1) \cdot B(a'+2b') \\ (8) \sum\limits_{\substack{i<r<\min(B'_{(i,j)}, b_{(i,j)}) \\ bp_G(r)=0}} Z_{WI'}(i+1, r-1) \cdot Z_{VP^R}(r, j-1) \cdot B(a'+2b') \\ (9) \sum\limits_{\substack{max(B_{(i,j)}, b'_{(i,j)})<r<j \\ bp_G(r)=0}} Z_{VP^L}(i+1, r) \cdot Z_{WI'}(r+1, j-1) \cdot B(a'+2b') \end{cases} \quad \text{(v)}$$

1.4.1. $i.bp(i)$ in G' crosses base pair in G, $bp(i)$ not equal to j

$$Z_{VP^R}(i,j) = \sum \begin{cases} (1) \sum_{max(B_{(i,j)},b'_{(i,j)})<r<j} Z_{VP}(i,r) \cdot Z_{WI'}(r+1,j) \\ (2) \text{ if empty}(G,[(r+1),j]) \\ \quad \sum_{max(B_{(i,j)},b'_{(i,j)})<r<j} Z_{VP}(i,r) \cdot B(c'(j-r)) \end{cases} \tag{vi}$$

1.4.2. $bp(j).j$ in G' crosses base pair in G, $bp(j)$ not equal to i

$$Z_{VP^L}(i,j) = \begin{array}{l} \text{if empty}(G,[i,(r-1)]) \\ \sum_{i<r<min(B'_{(i,j)},b_{(i,j)})} B(c'(r-i)) \cdot Z_{VP}(r,j) \end{array} \tag{vii}$$

1.5. $i.j$ closes a multiloop

$Z_{VM}(i,j)$ is the partition function over all structures $R_{i,j}$ for region $[i,j]$, if $[i,j]$ is weakly closed and $i.j$ closes a multiloop. Otherwise, $Z_{VM}(i,j) = 0$

$$Z_{VM}(i,j) = \sum \begin{cases} (1) \sum_{(i+1)<r\leq(j-1)} Z_{WM}(i+1,r-1) \cdot Z_{WM^1}(r,j-1) \cdot B(a+b) \\ (2) \sum_{(i+1)<r\leq(j-1)} Z_{WM}(i+1,r-1) \cdot Z_{WM^P}(r,j-1) \cdot B(a+b+P_{sm}) \\ (3) \sum_{i<r<(j-1)} B(c(r-i-1)) \cdot Z_{WM^P}(r,j-1) \cdot B(a+b+P_{sm}) \end{cases} \tag{viii}$$

1.6. i and j on a multiloop.

$Z_{WM}(i,j)$ is the partition function over all structures $R_{i,j}$ for region $[i,j]$, if $[i,j]$ is weakly closed, not empty, and i and j are on a multiloop. For base case where $i \geq j$, $Z_{WM}(i,j) = 0$.

$$Z_{WM}(i,j) = \sum \begin{cases} (1) \sum_{i\leq r<j} B(c(r-i)) \cdot Z_V(r,j) \cdot B(b) \\ (2) \sum_{i\leq r<j} B(c(r-i)) \cdot Z_P(r,j) \cdot B(b+P_{sm}) \\ (3) \sum_{i<r<(j-1)} Z_{WM}(i,r) \cdot Z_V(r+1,j) \cdot B(b) \\ (4) \sum_{i<r<(j-1)} Z_{WM}(i,r) \cdot Z_P(r+1,j) \cdot B(b+P_{sm}) \\ (5) \quad Z_{WM}(i,j-1) \cdot B(c) \end{cases} \tag{ix}$$

1.6.1. i and j on a multiloop, terminal stem pseudoknot-free

$Z_{WM^1}(i,j)$ is the partition function over all structures $R_{i,j}$ for region $[i,j]$, if $[i,j]$ is weakly closed, not empty, and i and j are on a multiloop (terminal stem, cf. Fig. 6). With Case (1), $i.j$ form a pseudoknot-free loop, and Case (2), j is unpaired. For base case where $i \geq j$, $Z_{WM^1}(i,j) = 0$.

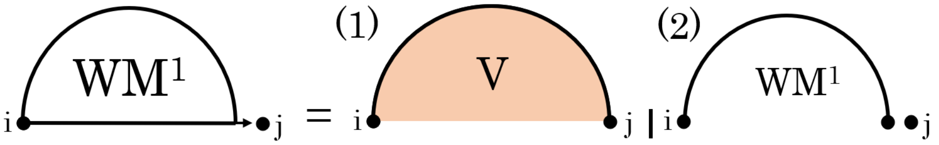

Fig. 6: Cases of WM^1. (1) terminal stem pseudoknot-free, (2) j is unpaired.

$$Z_{WM^1}(i,j) = \sum \begin{cases} (1) \quad Z_V(i,j) \cdot B(b) \\ (2) \quad Z_{WM^1}(i,j-1) \cdot B(c) \end{cases} \tag{x}$$

1.6.2. i and j on a multiloop, terminal stem pseudoknotted

$Z_{WM^P}(i,j)$ is the partition function over all structures $R_{i,j}$ for region $[i,j]$, if $[i,j]$ is weakly closed, not empty, and i and j are on a multiloop (pseudoknotted terminal branch, cf. Fig. 7). With Case (1), $i.j$ form a pseudoloop, and Case (2), j is unpaired. For base case where $i \geq j$, $Z_{WM^P}(i,j) = 0$.

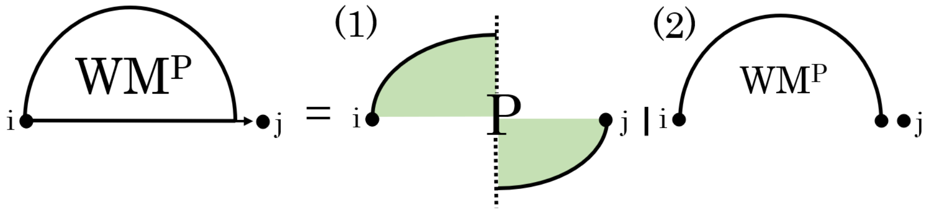

Fig. 7: Cases of WM^P. (1) terminal stem pseudoknotted, (2) j is unpaired.

$$Z_{WM^P}(i,j) = \sum \begin{cases} (1) \quad Z_P(i,j) \cdot B(b + P_{sm}) \\ (2) \quad Z_{WM^P}(i,j-1) \cdot B(c) \end{cases} \tag{xi}$$

1.7. Weakly closed subregions inside pseudoloop

$Z_{WI}(i,j)$ is the partition function over all structures $R_{i,j}$ given that $[i,j]$ is weakly closed ($cover(i) = cover(j) \neq 0$), and $R_{i,j}$ is inside a pseudoloop. If $i = j$ and $bp_G(i) = 0$, $[i,j]$ is empty and $Z_{WI}(i,j) = P_{up}$, i.e., penalty for unpaired base in a pseudoloop. $Z_{WI}(i,j) = 0$, if $i > j$. Otherwise, $Z_{WI}(i,j) = 0$ ($cover(i) \neq cover(j)$, subregion not weakly closed). Z_{WI} is similar to Z_W with additional penalties for base pair, unpaired bases, and pseudoknot initiation inside a pseudoloop.

$$Z_{WI}(i,j) = \sum \begin{cases} (1) \text{ if } r.j \in G, \text{or } (bp_G(r) = 0 \text{ and } bp_G(j) = 0) \\ \quad \sum\limits_{i \leq r < j} Z_{WI}(i,r-1) \cdot Z_V(r,j) \cdot B(P_{ps}) \\ (2) \sum\limits_{i \leq r < j} Z_{WI}(i,r-1) \cdot Z_P(r,j) \cdot B(P_{ps} + P_{sp}) \\ (3) \quad Z_{WI}(i,j-1) \cdot B(P_{up}) \end{cases} \tag{xii}$$

1.8. Non-empty weakly closed subregion inside band

$Z_{WI'}(i,j)$ is the partition function over all nonempty structures $R_{i,j}$, if $[i,j]$ is weakly closed with respect to G, given that $R_{i,j}$ is inside a band. Otherwise, $Z_{WI'}(i,j) = 0$. $Z_{WI'}$ is similar to Z_{WI} with additional penalties for base pair or unpaired base in multiloop spanning a band, and pseudoknot initiation inside a multiloop.

$$Z_{WI'}(i,j) = \sum \begin{cases} (1) \quad Z_V(i,j) \cdot B(b') \\ (2) \quad Z_P(i,j) \cdot B(b' + P_{sm}) \\ (3) \text{ if } r.j \in G, \text{or } (bp_G(r) = 0 \text{ and } bp_G(j) = 0) \\ \quad \sum\limits_{i < r < j} Z_{WI'}(i,r-1) \cdot Z_V(r,j) \cdot B(b') \\ (4) \sum\limits_{i < r < j} Z_{WI'}(i,r-1) \cdot Z_P(r,j) \cdot B(b' + P_{sm}) \\ (5) \text{ if } r.j \in G, \text{or } (bp_G(r) = 0 \text{ and } bp_G(j) = 0) \\ \quad \sum\limits_{i < r < j} B(c'(r-i)) \cdot Z_V(r,j) \cdot B(b') \\ (6) \sum\limits_{i < r < j} B(c'(r-i)) \cdot Z_P(r,j) \cdot B(b' + P_{sm}) \\ (7) \text{ if } bp_G(j) = 0 \\ \quad Z_{WI'}(i,j-1) \cdot B(c') \end{cases} \tag{xiii}$$

1.9. $i.j$ close a loop

$Z_V(i,j)$ is the partition function over all structures $R_{i,j}$ for region $[i,j]$, if $[i,j]$ is weakly closed or empty and $i.j$. Otherwise $Z_V(i,j) = 0$. This subfunction and $Z_{VBI}(i,j)$ to follow are unchanged from Mathews et al. (Mathews et al., 1999) pseudoknot-free algorithm, i.e., penalties for hairpin loop, stacked base pairs, internal/bulge loop, or multiloop.

1.9.1. $i.j$ close an internal/bulge loop

$Z_{VBI}(i,j)$ is the partition function over all structures $R_{i,j}$ for region $[i,j]$, if $[i,j]$ is weakly closed or empty and $i.j$ closes a bulge or internal loop. Otherwise $Z_{VBI}(i,j) = 0$ (Mathews et al., 1999).

1.10. $[i,i'] \cup [bp(i'), bp(i)]$ band region

$Z_{BE}(i,i')$ is the partition function over the band $[i',i] \cup [bp_G(i), bp_G(i')]$, if $i \leq i' < bp_G(i') \leq bp_G(i)$; otherwise $Z_{BE}(i,i') = 0$. In Case (1), $bp(i+1).bp(i)-1$ form a stacked pair in G. In Case (2), $i.bp(i)$ and $l.bp(l)$ in G close an internal loop. In Case (3), $[i+1, l-1]$ and $[bp(l)+1, bp(i)-1]$ are both weakly closed non-empty region. In Case (4), $[i+1, l-1]$ is a weakly closed non-empty region and $[bp(l)+1, bp(i)-1]$ is empty. In Case (5), $[bp(l)+1, bp(i)-1]$ is a weakly closed non-empty region and $[i+1, l-1]$ is empty (cf. Fig. 8). For base case, $Z_{BE}(i,i) = 0$ if $i < bp_G(i)$.

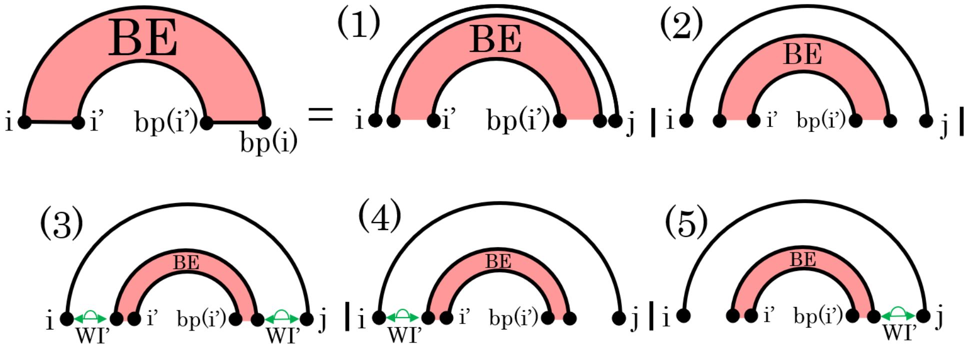

Fig. 8: Cases of BE. (1) stacked pair in G; (2) internal loop; (3) initial and terminal weakly closed non-empty regions. (4) initial weakly closed non-empty region and terminal loop. (5) initial loop and terminal weakly closed non-empty region.

$$Z_{BE}(i,i') = \sum \begin{cases} (1)\text{ if } bp_G(i+1) = bp_G(i)-1 \\ \quad B(e_{stP}(i, bp_G(i))) \cdot Z_{BE}(i+1, i') \\ (2)\text{ if } bp_G(l) > 0,\ \text{empty}(G, [i+1, l-1]),\ \text{empty}(G, [bp_G(l)+1, bp_G(i)-1]), \\ \quad\quad i < l \leq i',\ \text{and } (bp_G(i') \leq bp_G(l) < bp_G(i)) \\ \quad B(e_{intP}(i, l, bp_G(l), bp_G(i))) \cdot Z_{BE}(l, i') \\ (3)\text{ if } bp_G(l) > 0,\ \text{weakly closed}(G, [i+1, l-1]), \\ \quad\quad\quad \text{weakly closed}(G, [bp_G(l)+1, bp_G(i)-1]), i < l \leq i',\ \text{and } bp_G(i') \leq bp_G(l) < bp_G(i) \\ \quad Z_{WI'}(i+1, l-1) \cdot Z_{BE}(l, i') \cdot Z_{WI'}(bp_G(l)+1, bp_G(i)-1) \cdot B(a' + 3b') \\ (4)\text{ if } bp_G(l) > 0, \text{weakly closed}(G, [i+1, l-1]),\ \text{and empty}(G, [bp_G(l)+1, bp_G(i)-1]), \\ \quad\quad i < l \leq i',\ \text{and } bp_G(i') \leq bp_G(l) < bp_G(i) \\ \quad Z_{WI'}(i+1, l-1) \cdot Z_{BE}(l, i') \cdot B(a' + 2b' + c'(bp_G(i) - bp_G(l) + 1)) \\ (5)\text{ if } bp_G(l) > 0,\ \text{empty}(G, [i+1, l-1]), \text{weakly closed}(G, [bp_G(l)+1, bp_G(i)-1]), \\ \quad\quad i < l \leq i',\ \text{and } bp_G(i') \leq bp_G(l) < bp_G(i) \\ \quad B(a' + 2b' + c'(l-i-1)) \cdot Z_{BE}(l, i') \cdot Z_{WI'}(bp_G(l)+1, bp_G(i)-1) \end{cases} \quad \text{(xiv)}$$

References

M. S. Andronescu, C. Pop, and A. E. Condon. Improved free energy parameters for RNA pseudoknotted secondary structure prediction. *RNA*, 16(1):26–42, 2010.

H. Jabbari, A. Condon, and S. Zhao. Novel and Efficient RNA Secondary Structure Prediction Using Hierarchical Folding. *J. Comput. Biol.*, 15(2):139–163, Mar. 2008. doi: 10.1089/cmb.2007.0198.

D. H. Mathews, J. Sabina, M. Zuker, and D. H. Turner. Expanded sequence dependence of thermodynamic parameters improves prediction of RNA secondary structure. *J. Mol. Biol.*, 288(5):911–940, 1999.

B. Rastegari and A. Condon. Parsing nucleic acid pseudoknotted secondary structure: algorithm and applications. *J. Comput. Biol.*, 14(1):16–32, 2007.

J. Ren, B. Rastegari, A. Condon, and H. H. Hoos. Hotknots: heuristic prediction of RNA secondary structures including pseudoknots. *RNA*, 11(10):1494–1504, 2005.

C. Witwer, I. L. Hofacker, and P. F. Stadler. Prediction of consensus RNA secondary structures including pseudoknots. *IEEE/ACM Trans. Comput. Biol. Bioinf.*, 1(2):66–77, 2004.
